# Natural iron-containing minerals catalyze the degradation of polypropylene microplastics: a route to self-remediation learnt from the environment

**DOI:** 10.1007/s11356-024-34120-0

**Published:** 2024-07-03

**Authors:** Andrea Corti, Enrico Mugnaioli, Antonella Manariti, Gabriele Paoli, Filippo Petri, Pier Francesco Maria Tersigni, Alessio Ceccarini, Valter Castelvetro

**Affiliations:** 1 Department of Chemistry and Industrial Chemistry, University of Pisa, Via G Moruzzi 13, 56124 Pisa, Italy; 2 CISUP - Center for the Integration of Scientific Instruments of the University of Pisa, Lungarno Pacinotti 43, 56126 Pisa, Italy; 3 Department of Earth Science, University of Pisa, Via S Maria 53, 56126 Pisa, Italy

**Keywords:** Polyolefin, Photodegradation, Thermal degradation, Transition metal, Polymer oxidation, Hydrocarbon chain scission

## Abstract

Virgin and environmentally aged polypropylene (PP) micropowders (V-PP and E-PP, respectively) were used as reference microplastics (MPs) in comparative photo- and thermo-oxidative ageing experiments performed on their mixtures with a natural ferrous sand (NS) and with a metal-free silica sand (QS). The ferrous NS was found to catalyze the photo-oxidative degradation of V-PP after both UV and simulated solar light irradiation. The catalytic activity in the V-PP/NS mixture was highlighted by the comparatively higher fraction of photo-oxidized PP extracted in dichloromethane, and the higher carbonyl index of the bulk polymer extracted with boiling xylene, when compared with the V-PP/QS mixture. Similarly, NS showed a catalytic effect on the thermal degradation (at *T* = 60 °C) of E-PP. The results obtained indicate that, under suitable environmental conditions (in this case, an iron-containing sediment or soil matrix, combined with simulated solar irradiation), the degradation of some types of MPs could be much faster than anticipated. Given the widespread presence of iron minerals (including the magnetite and iron-rich serpentine found in NS) in both coastal and mainland soils and sediments, a higher than expected resilience of the environment to the contamination by this class of pollutants is anticipated, and possible routes to remediation of polluted natural environments by eco-compatible iron-based minerals are envisaged.

**Graphical abstract:**

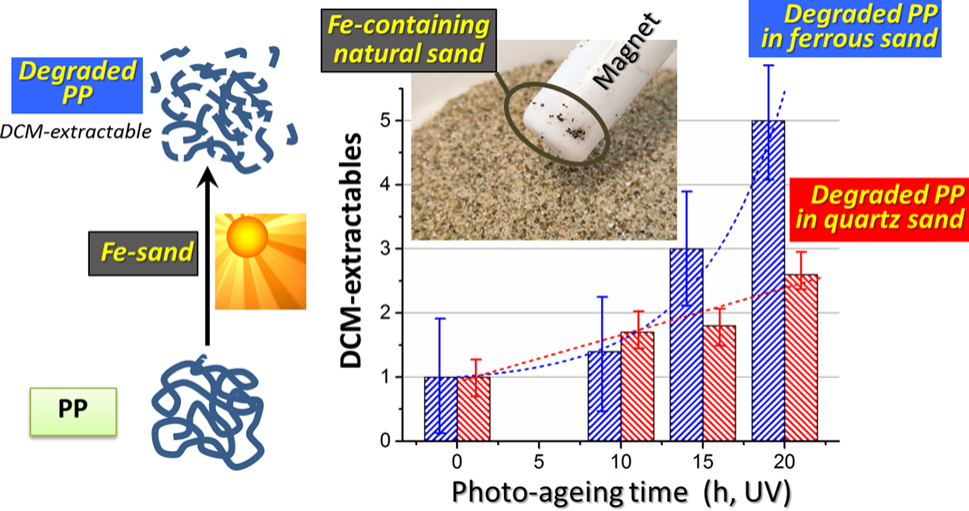

**Supplementary Information:**

The online version contains supplementary material available at 10.1007/s11356-024-34120-0.

## Introduction

The environmental pollution by plastics and their degradation products down to micro and nanoparticles, commonly referred to as microplastics (MPs) and nanoplastics (NPs), respectively, has become a worldwide emergency. An increasing number of studies is being devoted to developing methodologies for the analysis of MPs in various environmental matrices (Ivleva 2021; Dees et al. 2021), modeling their transport and distribution (van Sebille et al. 2020; Evangeliou et al. 2020), understanding the mechanisms and products of their environmental degradation (Liu et al. 2022; Min et al. 2020), exploring their effects on biological organisms (Charlton-Howard et al. 2023; Huang et al. 2021) and human health (WHO 2022). Concerning the proposed mitigation actions targeting prevention (Kasznik and Łapniewska 2023) and capture (Shen et al. 2021; Gkanasos et al. 2021) of MPs and NPs, these can hardly challenge the continuous increase of plastic waste entering the environment. However, the common notion of a nearly unlimited lifespan of plastics is a misconception (Sorasan et al. 2022). Depending on polymer type, plastic additives, item size and shape, and environmental exposure conditions, the degradation processes activated by photo-oxidation (the main degradation mechanism of fully carbon backbone polymers, such as polyolefins and vinyl polymers), and eventually hydrolytic and microbiological action, may greatly shorten the lifetime of synthetic polymers in the environment (Chamas et al. 2020). Such shorter lifetime is not only referred to the deceptive disappearance of MPs as they are progressively converted into elusive NPs, but also to macromolecular fragmentation into low molecular weight degradation products, resulting in mass loss due to the release of leachable or volatile species (Lomonaco et al. 2020; Biale et al. 2022; Ammala et al. 2011).

While the sources of MPs are extremely diverse, including textile fibers, tire wear particles, and peeled off coatings, the largest fraction of plastic waste dispersed in the environment arguably comes from packaging and other single use items. These are mainly made of low-density hydrocarbon polymers such as polyethylene (PE), polypropylene (PP), and polystyrene (PS), along with higher density heteropolymers (mainly polyester) (Erni-Cassola et al. 2019). Because of their low density, hydrocarbon polymer items float in water and, after reaching the oceans, they are likely to end up in coastal areas, and particularly beaches (Harris 2020). Thus, beaches become hot spots for the accumulation of MPs, either deposited as such or continuously generated upon ageing and fragmentation of larger plastic items (Sorasan et al. 2022).

**Fig. 1:**
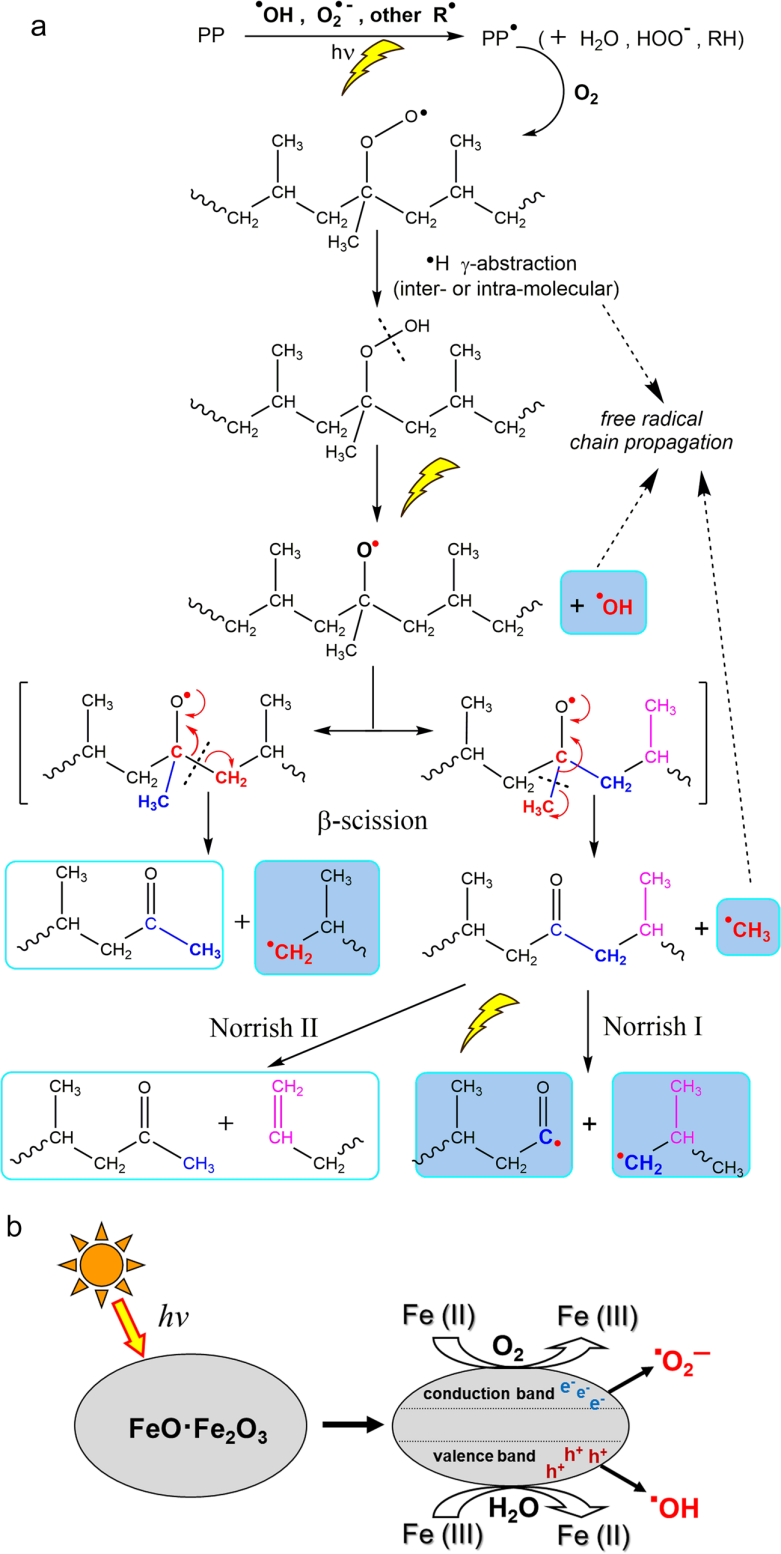
**A** PP photo-oxidative degradation pathways: H-abstraction (more likely on tertiary carbons), followed by oxygen pickup and further H-abstraction by the new peroxy-radical; the resulting thermo- and photo-labile hydroperoxide decomposes into oxy-radical, and eventually ketones upon various β-scissions mechanisms involving the main chain C–C bond; Norrish type reactions of ketones cause further main chain scissions, all the above generating new free radicals (highlighted in the figure), with cascade amplification of the degradation reactions. **B** Schematized photo-activated generation of ROS by hole (h^+^) or electron (e^−^) transfer from magnetite

The main mechanisms of photo-oxidation, and of the thermally-activated processes involving reactive species typically generated by photo-oxidation, are exemplified in Fig. 1A for PP, arguably the most readily photo-oxidized polyolefin among the fully carbon backbone polymers. They start with the generation of primary free radicals, typically as a result of photo- or thermally activated reactions activated by impurities (e.g., transition metal compounds, photosensitive species, reactive groups present as structural defects in the polymer); these can extract hydrogen atoms from the polymer by homolytic C-H cleavage, kicking off a combination of free radical chain reactions and of other photo- or thermo-activated processes. The former include oxygen pickup with formation of peroxy-radicals, chain fragmentation by β-scission of oxy-radicals, free radical transfer and coupling, among others. The latter include Norrish-type fragmentation of oxidized polymer chains, and other reactions of newly generated (oxidized) functional groups causing a cascade amplification of free radical generation (kinetic chain branching), etc. (Allen et al. 1985; Bertoldo et al. 2003; Fernando et al. 2007; Smith et al. 2018; Qiu et al. 2023).

The rate of polymer degradation may be further increased by the catalytic action of transition metals, which can interact with plastic items and MPs either upon adsorption from polluted environments or by contact with metal-containing minerals in the sediment or soil. Natural, semi-synthetic, or synthetic compounds such as the iron (hydr)oxides FeO(OH) (goethite) and α-Fe_2_O_3_ (hematite) (Ding et al. 2022), natural Fe-containing clays (Wang et al. 2023; Scaffaro et al. 2021), and a layered double hydroxide of MgAl modified with Ti and Cu (Jiang et al. 2023), have been reported to catalyze the photo- and thermo-oxidative degradation of various polymers (PE, PS, aliphatic polyesters). Indeed, several transition metal compounds, known to catalyze both photo- and thermo-activated degradation of polyolefins, are used as additives in the so-called oxo-biodegradable polyolefins (no longer allowed in the EU because they are considered sources of microplastics), to speed up polymer oxidation and arguably facilitate biodegradation (Montagna et al. 2015; Mamin et al. 2023).

The catalytic action of transition metal compounds may involve photo-generation of primary free radical species, along with thermal and photochemical decomposition of hydroperoxides (Hiatt et al. 1968) resulting from the above secondary oxidation reactions. From an energetic point of view, irradiation of various semiconducting oxides, including iron (hydr)oxides very common in environmental matrices, generates hole/electron couples in their valence/conduction bands (Ding et al. 2022). Once generated, these may transfer energy, electrons, or holes. In the first case, direct sensitization of the polymer, or of other organic or inorganic compounds present as impurities or plastic additives, may result in homolytic C-H cleavage, triggering the previously outlined chain of polymer degradation reactions (Rabek and Ranby 1975). In the other cases, electrons and holes may react with adsorbed H_2_O, OH^−^, and O_2_, generating reactive oxygen species (ROS, Fig. 1B) such as the superoxide radical anion (${\cdot O}_{2}^{-}$) and, as follow up reaction products, the hydroxyl radical ($\cdot OH$) and the highly reactive singlet oxygen (${}^{1}{O}_{2}$), according to Eq. 1–4:

1 $${O}_{2}+F{e}^{2+} \to { \cdot O}_{2}^{-}+F{e}^{3+}$$

2 $${\cdot O}_{2}^{-}+F{e}^{2+}+2{H}^{+} \to { {H}_{2}O}_{2}+F{e}^{3+}$$

3 $${{H}_{2}O}_{2}+F{e}^{2+} \to \cdot OH+H{O}^{-}+F{e}^{3+}$$

4 $${2 \cdot O}_{2}^{-}+2{H}^{+} \to {}^{1}{O}_{2}$$

Ultimately, all the above species can activate polymer degradation process (Uheida et al. 2021; Pino-Ramos et al. 2021). Generation of highly mobile and reactive free radicals may include, in addition to ROS, other infectious free radical species (Celina et al. 2006; Zhu et al. 2019) contributing to the overall degradation pattern.

The present study was prompted by the detection of surprisingly different concentrations of MPs in two sandy beaches located 2 km and 6 km, respectively, north of the Serchio river estuary in Tuscany, Italy. The total mass concentration of polyolefin (PE and PP) and PS MPs varied from 59–302 mg/kg sand across beach transects in Marina di Vecchiano (MV) (Ceccarini et al. 2018), while much lower values (always below 3 mg/kg) were measured in the unmanaged beach of Lecciona (LB) (Corti et al. 2023). Aside from a possible contribution to the higher MPs concentration in MV by the closer river estuary (Andriolo and Gonçalves 2022; Constant et al. 2019), the concentrations similarly low across the transects in LB was quite surprising. Indeed, the concentration of MPs is generally higher in the dunal area, a typical accumulation zone, than in the swash zone, subject to cyclical deposition and removal of MPs (Ceccarini et al. 2018). To explain such differences between the two beaches, some catalytic role of the sand matrix in LB, possibly related to the presence of minerals catalyzing photo- and thermo-oxidative polymer degradation processes, was postulated. Thus, a series of photo-ageing experiments was performed on a micropowder of virgin PP (V-PP) dispersed in two different sand matrices: the natural sand (NS) from LB, and a metal-free natural quartz sand (QS). Polypropylene was used as a test polymer because of its high content of tertiary C-H bonds, making it arguably the most reactive among polyolefins under photo-oxidative conditions, and thus allowing degradation to be assessed even after relatively short artificial ageing times (Grause et al. 2020; Ojeda et al. 2011; Lomonaco et al. 2020; Castelvetro et al. 2021b). Since NS was found to contain a relatively high concentration of paramagnetic minerals, a photo-ageing experiment was also carried out on V-PP dispersed in a NS sub-sample enriched in such minerals (NS_Fe_). Finally, the specific role of temperature in the catalytic degradation of PP was assessed by running thermal ageing experiments on a micropowder of environmentally aged PP (E-PP), and on V-PP as a reference, each one individually dispersed in both NS and QS. Differently from previous studies, in which the role of temperature had been investigated in combination with irradiation (François-Heude et al. 2015), here thermal ageing was performed in the dark.

## Materials and methods

### Test materials and environmentally aged samples

Environmental PP (E-PP) micropowder was obtained by cryomilling a set of bottle caps collected in LB (see the “Introduction” section). PP particles with nominal size < 500 μm were obtained using a ZM 200 Retsch ultracentrifugal mill with a 0.5 mm aperture size ring sieve. The absence of detectable amounts of additives was assessed by attenuated total reflectance (ATR) FT-IR spectroscopy. It is worth pointing out that such heterogeneous E-PP micropowder, with average oxidation level likely lower than that of true MPs, was a necessary choice since collecting true environmental MPs of a specific polymer type in an amount large enough for testing is not a feasible option.

A virgin PP (V-PP) micropowder produced by cryomilling (density 0.900 g/cm^3^, average particle size 857 μm, with 75 vol% particles in the 0.1–10 μm range) was a gift from Poliplast SpA (Casnigo, Italy).

A natural sand (NS) sample of about 1000 cm^3^ was collected with a glass bottle in the same area as the E-PP sample (geolocation 43.831447 N – 10.250860 E). The sample was sieved at 10 mm to remove larger debris of both natural and synthetic (e.g., plastic) origin, and then sequentially extracted 6 h with boiling dichloromethane (DCM) and xylenes (Xy) (HPLC grade solvents, Merck) to remove any polyolefin MPs and their degradation products (Castelvetro et al. 2021a). A sub-sample of NS enriched in iron-containing minerals, NS_Fe_, was obtained by manual separation using a magnet. The silica sand QS (Sea sand, particle size 0.100–0.315 mm, concentrated HCl solubles < 0.5%, Merck, Germany) was used as purchased.

### Experimental design

**Fig. 2:**
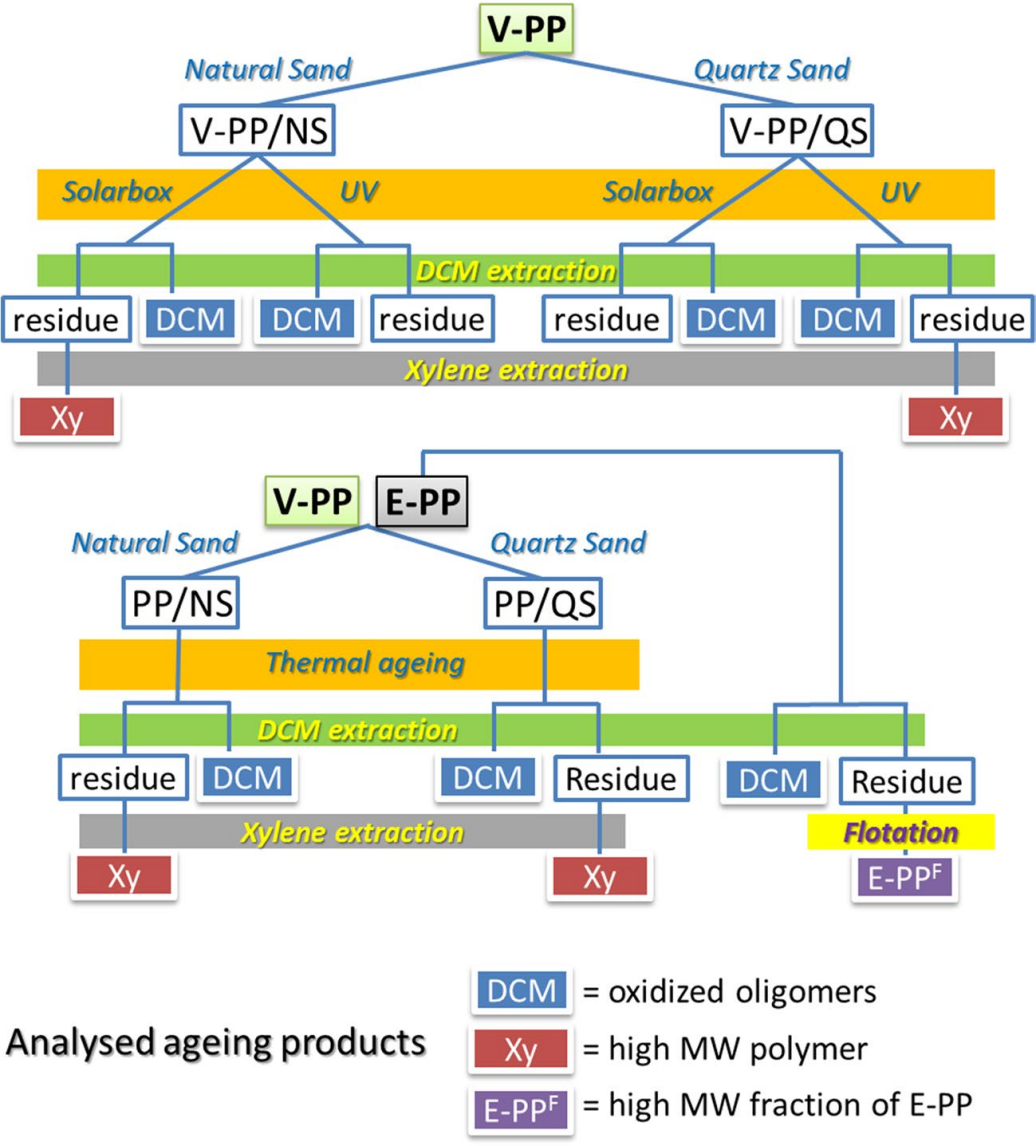
Experimental design (DCM and Xy extraction solvents, respectively; NS and QS natural ferrous sand and quartz sand, respectively; V-PP and E-PP micropowders of virgin and environmentally aged polypropylene, respectively)

The various accelerated ageing procedures by UV or simulated solar irradiation of V-PP and by thermal treatment of E-PP, along with the fractional solvent extraction procedures, are shown in Fig. 2 and described in the “Accelerated weathering tests” and the “Analytical characterization” sections.

### Accelerated weathering tests

Accelerated photo-ageing and thermal ageing experiments were performed on mixtures of V-PP or E-PP with the NS (V-PP/NS; E-PP/NS) and QS (V-PP/QS; E-PP/QS) sand, respectively, as described below.

#### Thermal degradation

NS and QS samples (10.0 g) were separately mixed with V-PP and E-PP powders (0.040 g) in 100 mL glass vials with Teflon-lined rubber stopper. The vials were then heated at 60 °C in a Binder ventilated oven; the number of vials was sufficient to allow withdrawal and subsequent analyses in triplicate after 35 days. Uncalibrated semi-quantitative analysis of the generated volatile degradation products was performed by sampling the vial headspace with SPME (solid phase microextraction), then by desorption/injection of the adsorbed substances directly in the gas-chromatograph (see the “Analytical characterization” section).

#### Photo-oxidative degradation

For the UV irradiation experiment 25 g of sand was thoroughly mixed with 0.100 g V-PP and transferred into a glass Petri dish with 9 cm diameter, resulting in a sand layer of about 1.5 cm height. The samples were further homogenized, to ensure that the V-PP particles were mostly buried in the sand layer, and irradiated with a Polymer 400 W medium-pressure mercury lamp (Helios Italquartz, Cambiago, Italy) at *E*_e(254 nm)_ = 13.4 W/cm^2^ and *E*_e(365 nm)_ = 18.4 W/cm^2^ (measured with a radiometer at the same 15 cm distance from the lamp as the Petri dish). Three samples of each V-PP/sand mixture were exposed to irradiation for 10, 15, and 20 h, respectively.

Simulated solar light photo-ageing were performed on V-PP/sand mixtures (0.100 g V-PP and 25.0 g sand) placed in 500 mL quartz tubes with polytetrafluoroethylene (PTFE) screw cap, and homogenized to avoid any excess of microplastics in the surface layer of the mixtures. Irradiation was performed at 40 °C (compatible with the summer temperature in the sampled beach) in a RH 3000e solarbox (COFOMEGRA, Milan, Italy) equipped with Xe lamp and outdoor filter, operated at 750 W/m^2^ intensity during 35 days. Such irradiation intensity roughly corresponds to fourfold the yearly-average in the sampling site (https://pvwattsnrelgov/), or over 140 days without considering the cloud screening, and very roughly to 5–10 h of UV lamp in the previous experiment, if one simply takes into account the intensity of the irradiation.

At the end of irradiation, the samples were extracted for 6 h with boiling DCM using a kumagawa continuous hot solvent extractor, to obtain the oligomeric and molecular fragments resulting from photo-oxidative degradation of the originally DCM-insoluble PP. The residues were further extracted for 6 h with boiling Xy, to quantitatively recover the less degraded PP. The DCM-extracted fraction was recovered by distilling off the solvent in a rotary evaporator, while the Xy extracts were obtained by partial evaporation of the solvent and cautious precipitation of the polymer by pouring the hot solution into a 20-fold excess methanol. All subsequent characterizations of the products were performed in triplicate. All samples and subsamples (PP micropowder, extracts, extraction residues) were kept in a refrigerator when not in use.

### Analytical characterization

Gravimetric analysis on solvent extracts, conditioned 7 h at 60 °C in a ventilated oven and allowed to cool at room temperature in a desiccator, was performed by weighing up to a constant weight (95% confidence by considering the pooled standard deviation) using a Kern ALS 220 − 4 balance (1·10^–5^ g precision).

Fourier Transformed Infrared Spectroscopy (FT-IR) spectra were recorded with a Nicolet iS50 spectrophotometer in the 4000–400 cm^−1^ range by collecting 32 transients at 4 cm^−1^ resolution. The soluble fractions were analyzed as films cast onto anhydrous KBr disks. Polymer residues after solvent extractions were ground with KBr in a mortar and converted into discs at 2000 psi.

Molecular weights and molecular weight distributions of the DCM-soluble oligomeric fractions of pristine and aged PP were determined by Size Exclusion Chromatography (SEC) using a Jasco PU-2089 Plus pump and injector, a column oven thermostated at 30 °C, RI-2031 Plus refractive index detector and UV-2077 Plus multiple wavelength UV/vis detector set at 260 and 340 nm. Analyses were performed using two in series LC columns (Phenogel 5 μm 500 Å 300 × 7.8 mm and Phenogel 5 μm 100 Å 300 × 7.8 mm), with chloroform eluent at 1.0 mL/min flow rate; after filtration of the DCM solutions through 0.2 μm PTFE filter membranes without detectable retention of insolubles; calibration was performed with a 0.8, 1.68, 2.4, 4.0, and 10.3 kDa set of polystyrene standards (Polymer Laboratories).

Gas-chromatography/mass spectrometry (GC/MS) analysis was performed on an Agilent 78908 instrument using the following setup: 1 mL/min He carrier gas; splitless injection with injector at *T* = 230 °C; DB 625 30 m column; triple quadrupole Agilent 7010 GC/MS Triple Quad detector operated in SCAN mode in the 30–300 m/z range. Temperature program: 5 min isothermal at 35 °C, then up to 260 °C at 5 °C/min, and final 5 min at 260 °C.

Thermogravimetric analyses (TGA) were performed on 5–10 mg samples using a Mettler Toledo TGA/SDTA 851e instrument at a 10 °C/min heating rate from 25 to 600 °C under dry nitrogen.

The metal content of the NS sand was determined by Inductively Coupled Plasma-Optical Emission Spectroscopy (ICP-OES) using a Thermo Fisher Scientific ICAP 7000 instrument. Solubilization of the sample was performed by microwave digestion in 85% nitric acid (US EPA Method 3051A), followed by dilution in 2% nitric acid for the analyses. Standard solutions of individual elements were used for calibration.

The mineralogical composition of the NS_Fe_ fraction (about 10 wt% of the total NS), obtained by coarse isolation of iron-rich particles from NS with the help of a magnet, was analyzed by scanning electron microscopy (SEM) and transmission electron microscopy (TEM). For the SEM analysis, the sample, placed on a stub covered with conductive carbon tape, was introduced in a FEI-Quanta 450 ESEM-FEG equipped with Schottky FEG source and energy-dispersive X-ray spectrometer (EDS) Bruker QUANTAX XFlash Detector 6|10, operated between 5 and 20 kV acceleration voltage. The sample for TEM observation was crushed with an agate mortar, dispersed in ethanol, sonicated for about 2 min, and then pipetted on a copper grid coated by amorphous carbon. Dark-field scanning TEM (DF-STEM), electron diffraction (ED), and energy-dispersive X-ray spectroscopy (EDS) were performed with a JEOL JEM-F2000 Multipurpose Electron Microscope equipped with a Schottky-FEG source and a SDD EDS detector, operated at 200 kV. Conventional selected area electron diffraction (SAED) and 3D electron diffraction (3DED) (Gemmi et al. 2019) data were recorded with an ASI Chetaah hybrid-pixel detector. 3DED data were collected stepwise with a tilt step of 1° and analyzed by the ADT3D software (Kolb et al. 2011).

## Results

The overall experimental design for the evaluation of a possible catalytic role of NS in the degradation of PP, including the different ageing procedures of PP/NS and PP/QS polymer/sand mixtures, and the separation of variously degraded polymer fractions by selective solvent extraction, is shown in Fig. 2 and detailed in the “Materials and methods” section. The ageing experiments were performed using PP/sand mixtures at concentrations in the order of 4–5 g/kg sand, quite higher than those typically found in polluted natural environments (Ceccarini et al. 2018). This was to ensure that enough polymeric material be available for the subsequent characterizations, while preventing PP MPs agglomeration and granting a large enough PP/sand interface area.

### Characterization of PP polymeric materials and natural sand matrices

**Fig. 3:**
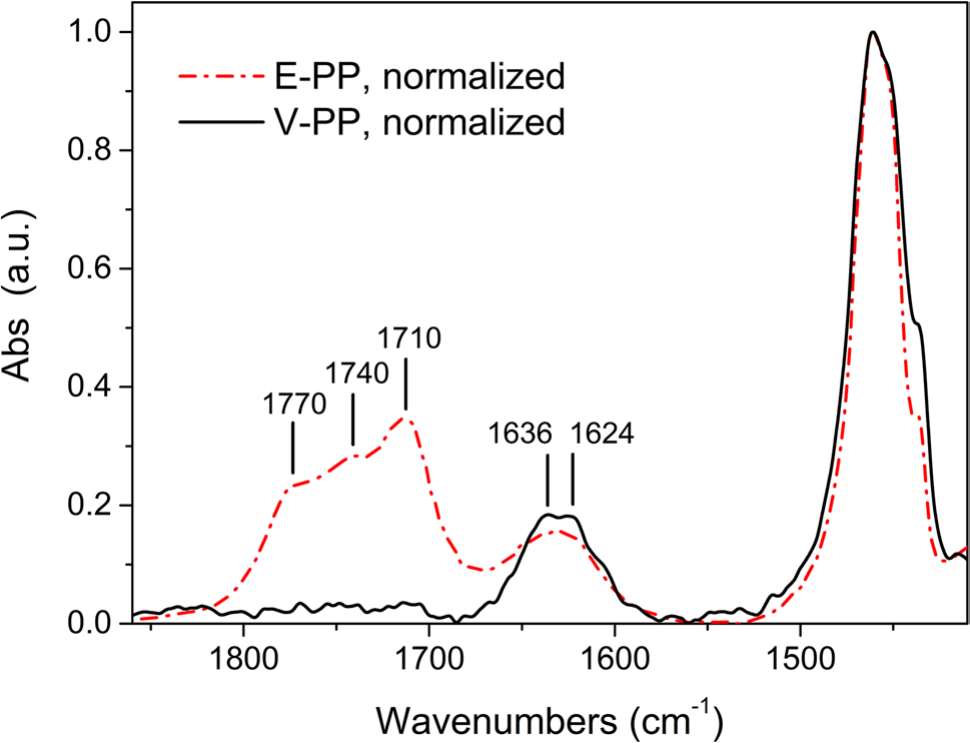
Carbonyl region in the FT-IR spectra of V-PP and E-PP; spectra normalized vs. the methylene bending absorption at 1462 cm.^−1^

Triplicate DCM extractions of the two starting PP micropowders, V-PP and E-PP, gave 1.1 ± 0.3 wt% and 7.0 ± 1.2 wt% of dry extract, respectively. In the case of V-PP, the oligomeric fraction is most likely the result of some thermo-mechanical degradation caused by the industrial cryomilling processing to obtain the micropowder used in this work. The FTIR spectra of the two polymers present significant differences in the carbonyl region between 1800 and 1700 cm^−1^ (Fig. 3), along with some differences in the O–H stretching zone at 3600–3250 cm^−1^ (possible contributions from carboxylic acid, hydroxy, and hydroperoxy groups) (Lamaire et al. 1983) and in the aliphatic C-H deformation zone between 1480 and 1350 cm^−1^. The broad and structured absorption from different carbonyl species in E-PP, indicating environmental oxidation, are absent in the spectrum of V-PP, presenting absorptions at 1620–1640 cm^−1^ attributed to chain end vinyl groups. The latter may result from disproportionation of free radicals generated during polymer synthesis (chain termination), and possibly during the cryomilling, causing homolytic C–C bond cleavage. The structured carbonyl absorption of E-PP presents peaks from conjugated ketone (1693 and 1682 cm^−1^), carboxylic acid dimers (1700–1715 cm^−1^), ketone (about 1720 cm^−1^), ester and peroxy acid (1730–1740 cm^−1^), peroxy ester and/or lactone and/or free acid groups (1750–1770 cm^−1^) (Bertoldo et al. 2003). The extent of oxidation of hydrocarbon polymers is usually quantified by considering the carbonyl index, CI, although its accuracy has been questioned by some authors (Rouillon et al. 2016). Here, values of CI = 0.78 for E-PP, and CI≈0 for V-PP, were obtained as the ratios between the integral of the cumulative carbonyl stretching absorption between 1830 and 1660 cm^−1^, and that of the methylene deformation between 1530 and 1415 cm^−1^.

The content of the main metal elements in NS, determined by duplicate ICP analyses, was Ni = 265 ppm, Al = 4662 ppm, Fe = 8956 ppm, Mn = 584 ppm, and Zn = 26 ppm (ppm = mg/kg). A dark colored component of NS, from which about 10 wt% of a NS_Fe_ fraction could be isolated using a magnet, turned out to be mainly composed of iron-containing paramagnetic particles.

**Fig. 4:**
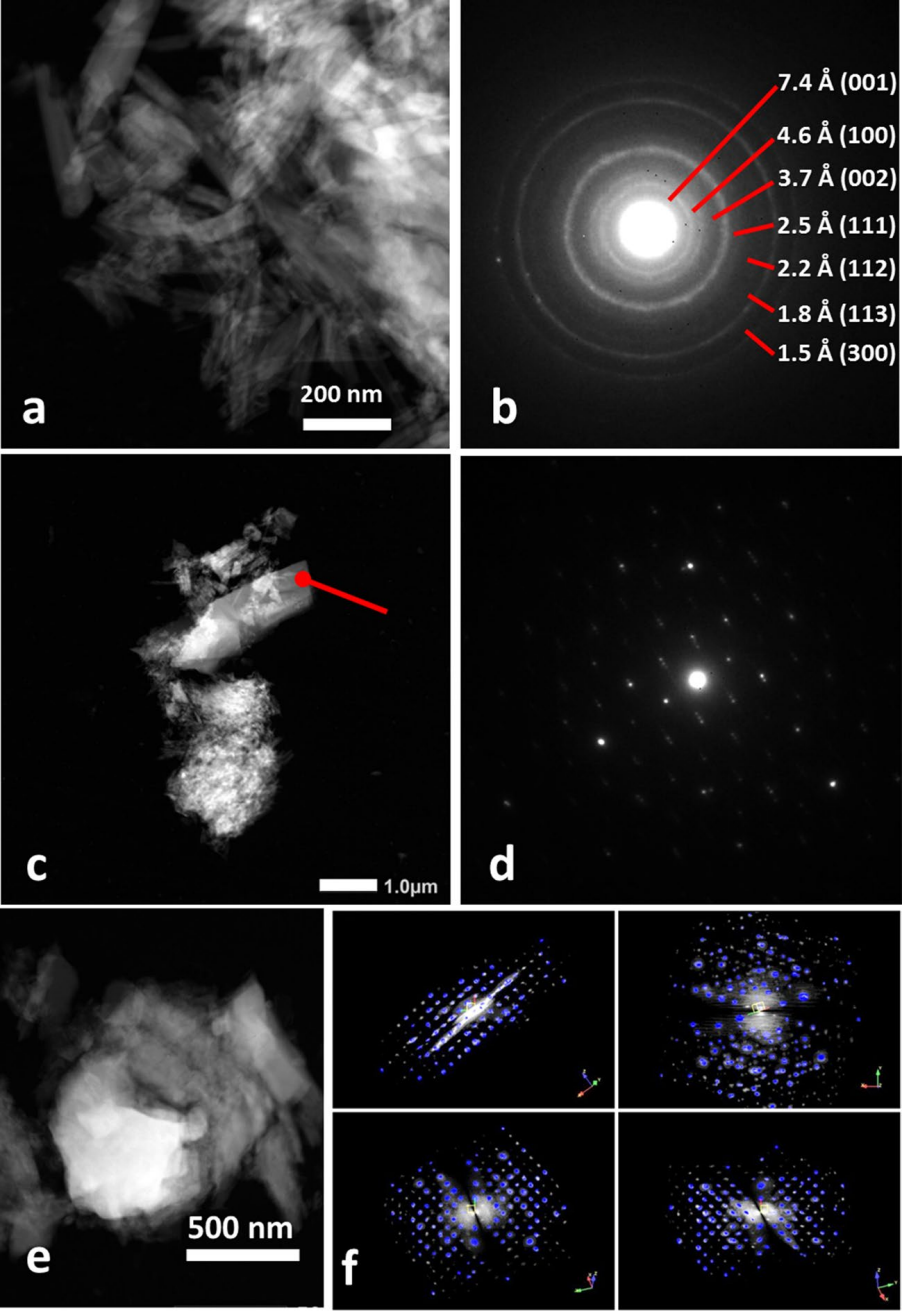
TEM analysis of NS_Fe_ showing a composition of serpentine polymorphs and magnetite: **a** chrysotile fibers with 13.5% Fe; **b** electron diffraction pattern of polycrystalline area in **a**; **c** modulated antigorite with 3.84% Fe; **d** selected area electron diffraction (SAED) of the spot highlighted in **c**; **e** magnetite, Fe_3_O_4_ (76% Fe, close to stoichiometric 72%); **f** 3D electron diffraction of the cubic face-centered cell of magnetite, with *a* = 8.4 Å

Characterization of the NS_Fe_ fraction by TEM analysis (see some results in Fig. 4) allowed to identify, from the electron diffraction pattern, most of the minerals (about 90% of those deposited on the grid) as polymorphs of the serpentine group, a phyllosilicate with base composition Mg_3_Si_2_O_5_(OH)_4_. All three serpentine polymorphs, namely lamellar lizardite, antigorite, and fibrous chrysotile, were detected. Serpentine minerals may contain other metal ions as isomorph substituents of Mg^2+^; among them, Fe^2+^ and Fe^3+^ (Evans et al. 2013), which is consistent with single particle compositions containing Fe typically in the 3–7 wt% range in the analyzed fraction. A few particles of iron oxides were also identified, and among them magnetite (Fe_3_O_4_). Isolated grains of calcite (CaCO_3_, often intergrown with serpentine minerals) and titanium dioxide (possibly from sun filter formulations or from decaying paints or plastics containing TiO_2_ as pigments) were also detected.

The elemental analysis performed by SEM–EDS on a larger—and thus more representative—NS_Fe_ sample showed a high heterogeneity, the main minerals being aluminosilicates with a high Mg (average slightly above 10 wt%) and Fe (average slightly below 10 wt%) content, along with relatively abundant serpentine of the chrysotile variety belonging to the asbestos fibers. The SEM–EDS results are in substantial agreement with the composition estimated by TEM. Single Fe–Cr alloy and Cu particles, most likely not of natural origin but deriving from waste produced by beach tourists, were also detected. The NS cumulative content of about 10 wt% of iron minerals (magnetite) and iron-containing silicates (Mg-Fe serpentine), the latter containing a roughly 10 wt% iron, results in an overall concentration of Fe^n+^ at least one order of magnitude higher than the 0.1 wt% or below typical of common silica sands.

### Accelerated ageing by UV and Xenon light irradiation

The two V-PP/NS and V-PP/QS mixtures were submitted to accelerated photo-ageing using either a UV lamp or a solarbox with a Xe lamp. The higher intensity and higher photon energy of the UV lamp generate higher instantaneous concentrations of free radical species, possibly resulting in additional reaction paths compared to the mechanisms activated with the Xe lamp. However, while the latter simulates more closely the environmental photo-oxidative ageing of polymers, the former may allow to identify long-term environmental exposure effects in much shorter lab experiments.

#### Accelerated photo-ageing by UV irradiation

**Fig. 5:**
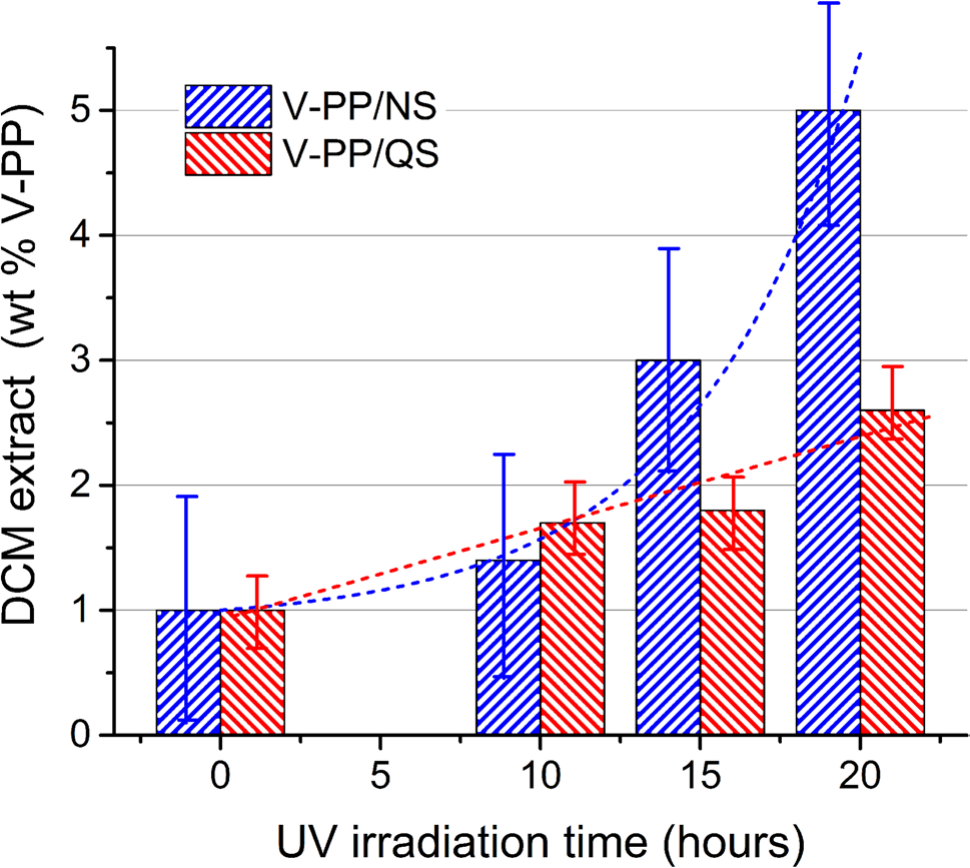
Time profile of DCM- extractable fraction from the V-PP/QS (blue bars) and V-PP/NS (red bars) mixtures after 20 h of UV irradiation, expressed as wt% of the total V-PP content; error bars are referred to triplicate experiments; dotted curves are the linear and exponential best fit for the DCM extracts over irradiation time, respectively

The dry weights of the DCM extracts, V-PP/NS_DCM_ and V-PP/QS_DCM_, increased with increasing irradiation time for both mixtures (Fig. 5 and Table 1). However, such increase was larger and nearly exponential for V-PP/NS_DCM_, compared to the slow and nearly linear increase for V-PP/QS_DCM_. FT-IR analyses of the DCM extracts confirmed a high oxidation level for both (Figure S1 in the Supplementary Material, SM), highlighted by the presence of the broad and structured carbonyl absorption (Figure S2 in SM) with maxima at 1767, 1737, and 1717 cm^−1^ assigned to peroxy acids or lactones, esters, and carboxylic acid groups, respectively. However, the higher carbonyl index of V-PP/NS_DCM_ (Table 1), the presence of a weak aldehyde C-H stretching peak at 2838 cm^−1^, the lower intensity of the symmetric methylene stretching at 2850 cm^−1^, and the presence of a broad O–H stretching absorption around 3450 cm^−1^ from hydroxy and possibly hydroperoxy groups (Lamaire et al. 1983), clearly indicated a higher oxidation than in V-PP/QS_DCM_.

**Table 1:** DCM-extractable (DCM extract) and DCM-insoluble/xylene extractable (Xy extract) fractions of virgin PP mixtures with NS (V-PP/NS) and QS (V-PP/QS) sand matrices, after UV (20 h) or Xenon lamp (35 days) photo-ageing

V-PP ageing	Sand matrix	DCM extract				Xy extract ^(d)^		
		wt%^(a)^	CI^(b)^	${\overline{M} }_{w}$^(c)^ (Da)	${\overline{M} }_{w}/{\overline{M} }_{n}$	CI	*T*_d_ onset^(e)^ (°C)	*T*_d_ 5%^(f)^ (°C)
UV (20 h)	QS	2.6 ± 0.7	2.73	1170	1.65	0.070	222	219
	NS	5.0 ± 1.7	3.75	1487	1.73	0.167	219	213
Xe (35 d)	QS	4.2 ± 1.7	3.39	2170	1.48	0.094	n.d	n.d
	NS	3.5 ± 2.1	5.05	2050	1.42	0.154	n.d	n.d

^(a)^The DCM-extractable V-PP fraction before irradiation (*t* = *t*_0_) was 1.0 wt% (see Fig. 5)

^(b)^At *t* = *t*_0_ was CI≈0

^(c)^Weight average molecular weight measured by SEC, referred to a calibration with polystyrene standards; the very small amount of DCM extractable fraction at *t* = *t*_0_ gave ${\overline{M} }_{w}$=754 Da and polydispersity index ${\overline{M} }_{w}/{\overline{M} }_{n}$=1.27

^(d)^PP recovery was quantitative for the not irradiated V-PP/NS and V-PP/QS samples and nearly quantitative (considering the fraction already removed in the DCM extraction) for the irradiated samples

^(e)^*T*_d_ onset at *t* = *t*_0_ was 226 °C; data not recorded for the Xe-irradiated samples

^(f)^*T*_d_ 5% at *t* = *t*_0_ was 224 °C; data not recorded for the Xe-irradiated samples

The results of the SEC analyses on the DCM extracts were consistent with the expected oligomeric composition, given the insolubility of high molecular weight PP in DCM. The slightly higher weight average molecular weight of V-PP/NS_DCM_ (${\overline{M} }_{w}$, Table 1) is in agreement with its higher value of CI and the higher DCM-extractable weight fraction. All the above indicate a more extensive oxidation, which promotes solubilization of longer oligomeric fragments owing to their higher content of polar oxidized groups.

However, it should be noted that the DCM extracts of the photo-aged V-PP/sand samples were only small fractions of the initial V-PP charge, irrespective of the irradiation source and sand matrix. Further extraction of the residue from the DCM extraction of the UV-irradiated V-PP/sand samples using boiling Xy, an effective solvent for high molecular weight PP, granted recovery of the remaining PP. Overall, polymer recovery was quantitative for the Xy extract from a control non-irradiated sample, and only slightly below the nominal values (≈95% as Xy extracts, plus the fraction extracted with DCM) for those subjected to 20 h of UV irradiation. The modest losses could be due to volatilization of oxidized molecular degradation products (Lomonaco et al. 2020) and/or to crosslinking by inter-macromolecular free radical coupling reactions (Rouillon et al. 2016).

The FT-IR spectra of the Xy extracts, V-PP/NS_Xy_ and V-PP/QS_Xy_, indicated, again, a higher oxidation of the former, based on the more intense carbonyl absorptions in the 1700–1800 cm^−1^ range (Figure S3–S5 in SM). The resulting CI = 0.167 for V-PP/NS_Xy_, higher than CI = 0.070 for V-PP/QS_Xy_ (and CI≈0 for the control), is in agreement with the previous results concerning the DCM extracts and the thermogravimetric analyses (TGA). The latter showed a larger reduction of both the onset degradation temperature (*T*_d_ onset) and the temperature at 5% weight loss (*T*_d_ 5%) for V-PP/NS_Xy_ compared to V-PP/QS_Xy_ (Table 1).

#### Simulated solar light photo-ageing under Xe lamp

The UV-ageing experiment consistently pointed at some catalytic activity of the NS matrix in the photo-oxidation of V-PP, and more specifically of Fe^n+^ as the largely most abundant transition metal in NS. However, spurious effects due to the high energy of the UV radiation, and to thermally activated processes due to overheating from the UV lamp, could not be ruled out. To exclude such unintended possible contributions, a parallel photo-ageing experiment was carried out by irradiating V-PP/NS and V-PP/QS with a Xenon lamp in solarbox at 40 °C.

After 35 days of photo-ageing, V-PP/NS_DCM_ and V-PP/QS_DCM_ gave similar DCM-extractable fractions, with a slightly lower amount for V-PP/NS_DCM_ that, on the other hand, showed a higher oxidation level as in the case of UV irradiation. Such apparently inconsistent results for NS_DCM_ could actually be explained by a not negligible loss of highly oxidized and volatile molecular fragments. The structured FTIR carbonyl absorptions of the two DCM extracts presented only slight differences: V-PP/QS_DCM_ was characterized by a higher fraction of carboxylic acid (1707 cm^−1^) and peroxy acid or lactone (1761 cm^−1^), while V-PP/NS_DCM_ showed a higher fraction of ester groups (1732 cm^−1^) (Figure S6 in SM). Also, the SEC analysis of the two DCM extracts gave quite similar results (Table 1). However, given the quite small DCM extractable fractions from the Xe-irradiated V-PP/sand samples, the Xy extracts, V-PP/NS_Xy_ and V-PP/QS_Xy_, provided useful complementary information. The FTIR spectra of these main fractions, consisting of less degraded polymer, confirmed an oxidation level of V-PP/NS_Xy_ higher (CI = 0.154) than that of V-PP/QS_Xy_ (CI = 0.094). Besides, the spectrum of the former presented additional ester and ketone carbonyl peaks at 1732 cm^−1^ and 1722 cm^−1^, a slightly lower intensity of the methylene symmetric stretching at 2850 cm^−1^, and aldehyde C-H stretching absorption at 2938 cm^−1^ as in the UV-irradiated V-PP/NS_Xy_.

### Enhanced photo-oxidative degradation of V-PP on a Fe-enriched NS bed

**Fig. 6:**
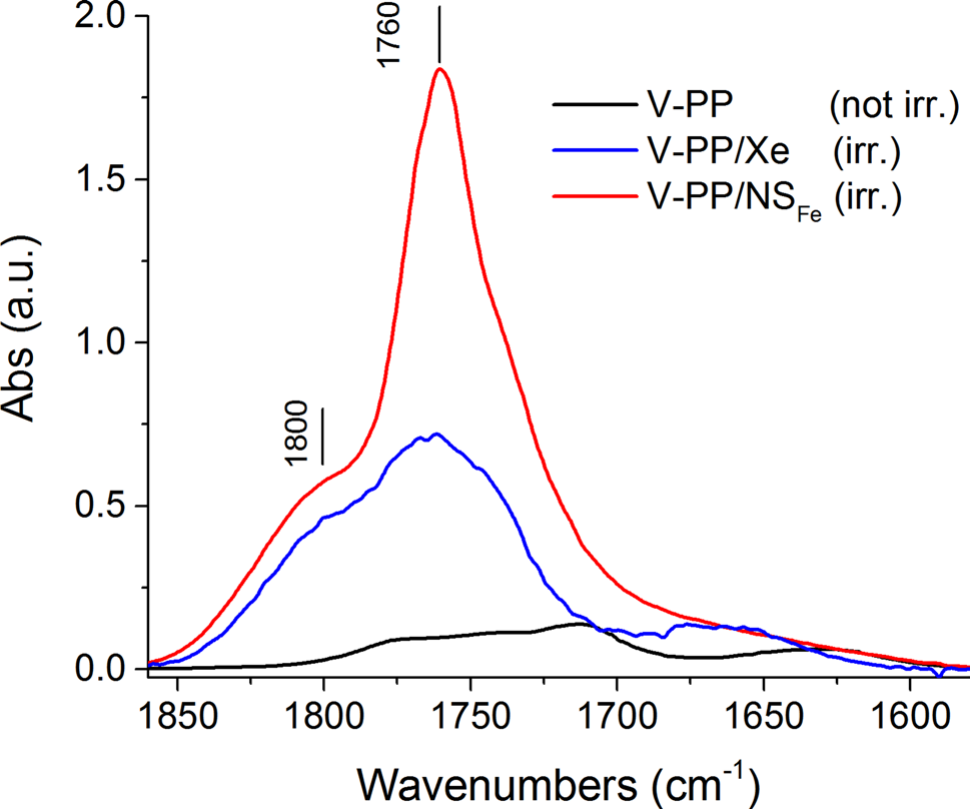
Carbonyl region in the FT-IR spectra of DCM extracts from V-PP irradiated 35 days in solar box: (i) V-PP blank (not irradiated, black line); (ii) V-PP irradiated without NS_Fe_ (blue line); (iii) V-PP irradiated in the presence of NS_Fe_ (red line)

The photo-catalytic effect of transition metal (Fe) minerals in the NS sand was further confirmed by the results of a photo-ageing experiment performed on a mixture of 0.100 g V-PP with 0.050 g of the iron-enriched NS_Fe_ (see the “Analytical characterization” and the “Characterization of PP polymeric materials and natural sand matrices” sections). The V-PP/NS_Fe_ mixture was placed in a spectrophotometric quartz cuvette and irradiated in the solar box in parallel with a second cuvette containing only V-PP as a control. The progress of photo-oxidation could already be observed after a few days of irradiation, causing the buildup of an opaque layer of waxy sublimation products on the walls of the cuvette. After 35 days of irradiation, PP degradation appeared to be significantly more pronounced in V-PP/NS_Fe_ (Figure S7 in SM), as confirmed by the higher oxidation and fragmentation of the DCM extracts measured by FT-IR and SEC analyses, respectively, and TGA analyses on the DCM-insoluble fractions. In Fig. 6, the FTIR spectra of the DCM extracts of the two irradiated samples present very intense carbonyl absorptions at 1760 cm^−1^ and around 1800 cm^−1^ from reactive peroxy acid and anhydride groups (Salvalaggio, et al. 2006), respectively, with additional acid, ester, and ketone/aldehyde groups contributing to the carbonyl envelope. The catalytic activity of the transition metal in V-PP/NS_Fe_ is clearly shown by the calculated CI = 15.96, about three times higher than the CI = 5.07 of the reference V-PP. The extensive photo-oxidation was confirmed by the SEC results (Figure S8 in SM), showing the presence of low oligomers with ${\overline{M} }_{w}$ of about 2.3–2.6 kDa in both DCM extracts, but a large fraction of high molecular weight (necessarily highly oxidized, to be soluble in DCM) PP only in V-PP/NS_Fe_. Differences were also observed in the less degraded residues from the DCM extractions, as the onset of thermal degradation (peak of the degradation rate) in the TGA thermograms was found to occur at lower temperature for V-PP/NS_Fe_ than for the control (V-PP irradiated without NS_Fe_). Such lower thermal stability is, again, indicative of a higher average oxidation of V-PP/NS_Fe_ (Figure S9 in SM).

### Thermal degradation tests on V-PP and on environmentally aged PP (E-PP)

To assess if the Fe-containing NS is also catalytically active in thermally activated PP degradation processes, thermal ageing tests were carried out in the dark on QS and NS mixtures with V-PP and with an environmentally aged PP micropowder (E-PP), respectively. The selected thermal ageing temperature of 60 °C compares well with the summer peak sand temperatures reached in LB (latitude 43° 50′ 19″ north), where the NS sand was sampled. After 35 days of thermal ageing, *t*(Δ) = 35, the extent of degradation was checked as before by analyzing the DCM extracts and the residual fraction extracted with refluxing Xy.

Contrary to the results of the photo-irradiation (whether UV or solarbox) experiments, the amounts and FTIR spectra of the DCM extracts from both thermally aged V-PP/sand samples remained nearly the same as those from the unaged sample at *t*(Δ) = 0 (Table 2). However, headspace GC/MS analysis revealed a more complex composition and comparatively higher concentrations (uncalibrated peak areas of typical PP degradation compounds, such as 2,4,4-trimethyl-1-pentene, 2-pentanone, 4-methyl-heptane and 2,4-dimethyl-heptene) of the mixture of volatile organic species for V-PP/NS than for V-PP/QS.

**Table 2:** Average CI values (from triplicate analyses) of the DCM extractable fractions upon thermal ageing at 60 °C of V-PP, E-PP, and their respective mixtures with QS and NS sands

Sample	Bed	*t*(Δ)^(a)^ (days)	DCM extract^(b)^ (wt%)	CI
V-PP	-	0	1.0	≈0
E-PP	-	0	7.1 ± 1.3	2.88
V-PP	NS	35	1.2 ± 0.3	0.34
V-PP	QS	35	1.2 ± 0.1	0.20
E-PP	NS	35	3.0 ± 1.8	2.33
E-PP	QS	35	3.3 ± 2.4	2.91

^(a)^Days of thermal ageing at 60 °C

^(b)^Weight % of the initial PP content

Differently from the results obtained with the V-PP/sand mixtures, both E-PP/sand mixtures were clearly affected by the thermal ageing. In particular, at *t*(Δ)_35_, they gave lower amounts of DCM extracts compared to the unaged E-PP. Besides, the FT-IR spectrum of the DCM extract from thermally aged E-PP/NS presented minor but noteworthy differences from those of both thermally aged E-PP/QS and unaged E-PP. In detail, while all three spectra show structured carbonyl absorptions with maxima at 1780, 1769, 1738, and 1713 cm^−1^ from lactone, peroxy acid, ester, and carboxylic acid groups (Figure S10 in SM) (Hiatt et al. 1975; Zahradničková et al. 1991), respectively, the overall intensity of the broad carbonyl band was lower in E-PP/NS than in E-PP/QS, with a corresponding lower CI value in the former (Table 2) in spite of its more distinctive yellowish appearance (Figure S11 in SM). Besides, the spectrum of the E-PP/NS extract was characterized by a comparatively lower intensity of the broad O–H stretching band around 3400 cm^−1^, and by the presence of weak additional peaks at 3015 (H-C = stretching), 1635 (C = C stretching), and 1409 (in-plane = C-H deformation) cm^−1^ (Figure S12 in SM).

To characterize the larger and less degraded PP fraction, the residues after thermal ageing and DCM extraction, E-PP^R^/QS and E-PP^R^/NS, two different procedures were used to isolate the polymeric fraction from the inorganic matrix: (i) extraction with boiling Xy, ensuring exhaustive recovery of high molecular weight PP; (ii) density separation using a saturated NaCl solution.

The Xy extraction yields were similar and slightly lower than quantitative (84 and 80 wt% in E-PP/NS and E-PP/QS, respectively), even when taking into account the fraction already extracted in DCM (Table 2). The FTIR spectra of the Xy extracts were quite similar (Figure S13 in the SM), with calculated CI = 0.15 for E-PP^R^/QS and CI = 0.14 for E-PP^R^/NS, lower than the CI = 0.23 for the Xy extract of (previously DCM-extracted) E-PP thermally treated without sand matrix.

By contrast, significantly different recovery rates and CI values of the polymer particulates, E-PP^R^/NS^F^ and E-PP^R^/QS^F^, were obtained as a result of the flotation procedures performed on the E-PP^R^/sand mixtures. In particular, the amount of E-PP^R^/NS^F^ was only 78% (standard deviation *σ* = 8%), against 94% (σ = 5%, both resulting from 6 replicate tests) for E-PP^R^/QS^F^, and about 100% for the unaged E-PP (the latter not extracted with DCM). The IR spectra had the usual general features of oxidized PP (Figure S14 in the SM), but the calculated CI = 0.65 for E-PP^R^/NS^F^ was over 50% higher than CI = 0.40 for E-PP^R^/QS^F^, although both apparently incongruously lower than that of the original E-PP powder with CI = 0.78.

## Discussion

In both UV and Xe lamp irradiation experiments, all the indicators (DCM extractable fraction, CI values from the FTIR spectra of both DCM extract and Xy extract) clearly pointed at a faster polymer degradation in V-PP/NS, suggesting a specific contribution by the iron minerals in NS. Such speculation was further confirmed by the extremely fast degradation of V-PP upon irradiation of its mixture with NS_Fe_, as noted in the “Enhanced photo-oxidative degradation of V-PP on a Fe-enriched NS bed” section (experiment not included in Fig. 2).

Thermal degradation of PP under the relatively mild environmental conditions is mainly the result of Arrhenius-like activation involving reactive functional groups (e.g., hydroperoxy), and increasing diffusivity of “infectious” free radical species, all of which being mainly the result of photo-oxidation and follow up reactions. This connection between photo-oxidative and thermally activated degradation was expected to be mirrored by a catalytic activity of the ferrous NS sand in both photo- and thermo-oxidative reactions.

In the thermal ageing experiments, the DCM-extractable (oligomeric) fraction from the E-PP/sand samples was found to be lower than that of the control unaged E-PP; besides, the DCM extract from E-PP/NS (CI = 2.33) showed an oxidation level lower than those from E-PP/QS (CI = 2.91) and from the untreated E-PP. Such results could be erroneously explained in terms of higher thermal stability of E-PP/NS, and lack of catalytic activity of the NS sand. On the contrary, they further support the hypothesis of a catalytic activity of the ferrous NS also in the thermo-oxidative degradation of PP. Indeed, the latter is kinetically hindered under the relatively mild environmental conditions, but it unlocks in the presence of thermally labile functional groups such as hydroperoxides, continuously generated during and after photo-oxidative ageing, and certainly present in E-PP. Their thermal decomposition, that is known to be catalyzed by Fe(II/III) and other transition metals compounds, triggers a cascade amplification of the concentration of free radicals, including the highly reactive oxyradical that can undergo β-cleavage involving main chain C–C bonds (Carlsson and Wiles 1969) (Fig. 2). Extensive main chain cleavage eventually results in the generation of low molecular weight (oxidized) fragments, easily lost as volatiles (Lomonaco et al. 2020). It is therefore reasonable to explain the lower amount and lower oxidation of the DCM-extractable fraction from E-PP/NS as the result of extensive loss of such fragments within the timeframe of the thermal ageing experiment. Indeed, such loss would remove the most highly degraded fraction of E-PP, which contributes most to the high CI and the DCM-extractables *before* the thermal treatment. Such speculation was supported by the presence, in the FTIR spectrum of the DCM extract of thermally treated E-PP/NS, of a broad absorption at about 1630 cm^−1^ from terminal C = C groups deriving from chain fragmentation through β-cleavage (Figure S12 in SM). In addition, the same extract displays a distinctive yellowish color (Figure S11 in SM), indicative of the presence of functional groups with absorption in the low wavelength range of the visible spectrum such as, e.g., α,β-conjugated carbonyls (Allen et al. 1985).

The main PP fraction, still present in the residues from DCM extraction of the E-PP^R^/sand samples, was recovered by the usual Xy extraction and, in a parallel experiment, by density separation. The Xy extraction procedure did not provide insightful information. The lower than quantitative recovery rates and low PP oxidation levels (even lower than that of V-PP) obtained, independent of the type of sand matrix, was ascribed to unintended further fragmentation and removal of oxidized molecular fragments due to the high extraction temperature (about 140 °C). On the other hand, the density separation yield of E-PP^R^/NS^F^ much lower than that of E-PP^R^/NS^F^, along with the higher oxidation level of the former, is indicative of a higher “stickiness” of E-PP^R^/NS^F^ due to more highly oxidized particle surface, resulting in less effective separation by flotation.

## Conclusions

The evidences of a catalytic role of metal-containing minerals, and in particular of magnetite and iron-containing serpentine, in the photo- and thermo-oxidative degradation of PP MPs, sheds a new light on the possible fate of microplastics polluting soils and coastal sediments. Such activity by iron minerals is potentially very relevant, since iron is a nearly ubiquitous element in natural soils and rocks, either within iron-containing minerals or as non-stoichiometric component. Serpentine minerals are relatively common in coastal areas and in major mountain ranges worldwide (Carmignano et al. 2020), from which rock erosion debris eventually end up into rivers and then feed as sediments the estuarine coastlines. Microplastic degradation processes similar to those highlighted in the present research are thus likely to occur not only in Fe-rich sandy beaches, but also in agricultural soils, where high concentrations of polyolefin MPs may result from agricultural practices (e.g., mulching films and greenhouse covers), involuntary introduction through wastewater treatment plants and industrial compost amendments, watering with polluted surface waters, and atmospheric transportation.

Several points need further investigation to accurately assess and quantify the relationship between the activity of iron (and of other catalytically active transition metal compounds) in the heterogeneous photo- and thermo-catalytic degradation of MPs, and the various factors that may affect such complex processes. Among the latter, the form and concentration of transition metal ions and of other ROS-generating species in the soil or sediment matrix, the environmental exposure conditions, and the possible contribution of microorganisms. However, the obtained results clearly indicate that the interaction of MPs with metal compounds in the environment is not limited to adsorption and transport of polluting metals *by* MPs, but may involve a favorable pro-degradant catalytic activity of metals *towards* MPs. In particular, while several transition metal compounds are known to catalyze the degradation of various polymers when embedded in the polymer matrix (as in the oxo-biodegradable polyolefins), our results have shown that an intimate polymer-metal contact is not a strict requirement, since such catalytic activity can also be displayed in the case of mineral-polymer particle loose mixtures. In our experiments, polymer-metal contact was much more limited than in the case of MPs with adsorbed metal ions (e.g., from polluted environments). This suggests that highly reactive and mobile ROS and/or other “infectious” reactive molecular fragments, generated by photo- and/or thermally activated mineral particles either directly or as a result of their catalytic activity, may play a key role in the overall degradation process, strongly enhancing the degradation rate governed by cascade free radical reactions.

In the end, the obtained results indicate that, depending on the environmental exposure conditions and environmental matrix, the environmental persistence of polyolefins, and likely of other kinds of MPs, may be much shorter than anticipated. This envisages a better environmental resilience to the ever-increasing plastics pollution, and provides useful suggestions concerning possible routes to develop sustainable and affordable remediation strategies for the polluted environment.

## Supplementary Information

Below is the link to the electronic supplementary material.

Supplementary file1 (DOCX 2407 KB)

## Data Availability

The authors declare that the data supporting the findings of this study are available within the paper and its Supplementary Information files. Should any raw data files be needed in another format they are available from the corresponding author upon reasonable request.
