# Supplementary material for: Natural iron-containing minerals catalyze the degradation of polypropylene microplastics: a route to self-remediation learnt from the environment
Source: Environ Sci Pollut Res Int. 2024 Jul 3;31(32):45162–76. doi: 10.1007/s11356-024-34120-0 (PMC11512828; doi:10.1007/s11356-024-34120-0)
Supplement: Supplementary file 1 — Supplementary file1 (DOCX 2407 KB) [file 11356_2024_34120_MOESM1_ESM.docx]

**Supplementary Material**

**Natural iron-containing minerals catalyze the degradation of polypropylene microplastics: a route to self-remediation learnt from the environment**

Andrea Corti^a,b^, Enrico Mugnaioli^b,c^, Antonella Manariti^a,b^, Gabriele Paoli^b^, Filippo Petri^,a^, Pier Francesco Maria Tersigni^a^, Alessio Ceccarini^a^, Valter Castelvetro^a,b,^*

*andrea.corti@unipi.it; enrico.mugnaioli@unipi.it; antonella.manariti@unipi.it; gabriele.paoli@unipi.it; p.tersigni@studenti.unipi.it; alessio.ceccarini@unipi.it; valter.castelvetro@unipi.it*

^a^ Department of Chemistry and Industrial Chemistry, University of Pisa, via G. Moruzzi 13, 56124 Pisa, Italy

^b^ CISUP - Center for the Integration of Scientific Instruments of the University of Pisa, Lungarno Pacinotti 43, 56126 Pisa, Italy

^c^ Department of Earth Science, University of Pisa, via S. Maria 53, 56126 Pisa, Italy

***** Correspondence: valter.castelvetro@unipi.it; Tel.: +39-0502219256

Department of Chemistry and Industrial Chemistry, via G. Moruzzi 13, 56124 Pisa, Italy

The carbonyl region of the FT-IR spectra from the UV-irradiated V-PP/QS and V-PP/NS, and the appearance of a broad absorption in the hydroxyl stretching region around 3400-3500 cm^-1^ indicate a high level of oxidation of DCM extracts of the UV-irradiated V-PP, and in particular of the DCM extracts from of V-PP/NS.





**Figure S1.** FT-IR spectra of the DCM extracts from of V-PP/QS and V-PP/NS after 20 h UV irradiation. Broad O-H stretching absorption from hydroxy, carboxylic acid, and hydroperoxy functional groups centered at about 3440 cm^-1^; asymmetric / symmetric methyl C-H stretching at 2950 / 2860 cm^-1^ and asymmetric / symmetric methylene C-H stretching at 2950 / 2860 **cm**^-1^; aldehyde asymmetric C-H stretching at 2838 cm^-1^; structured carbonyl stretching with lactone/peroxyacid (1760 cm^-1^) and conjugated ketone/carboxylic acid (1715 cm^-1^) peaks.


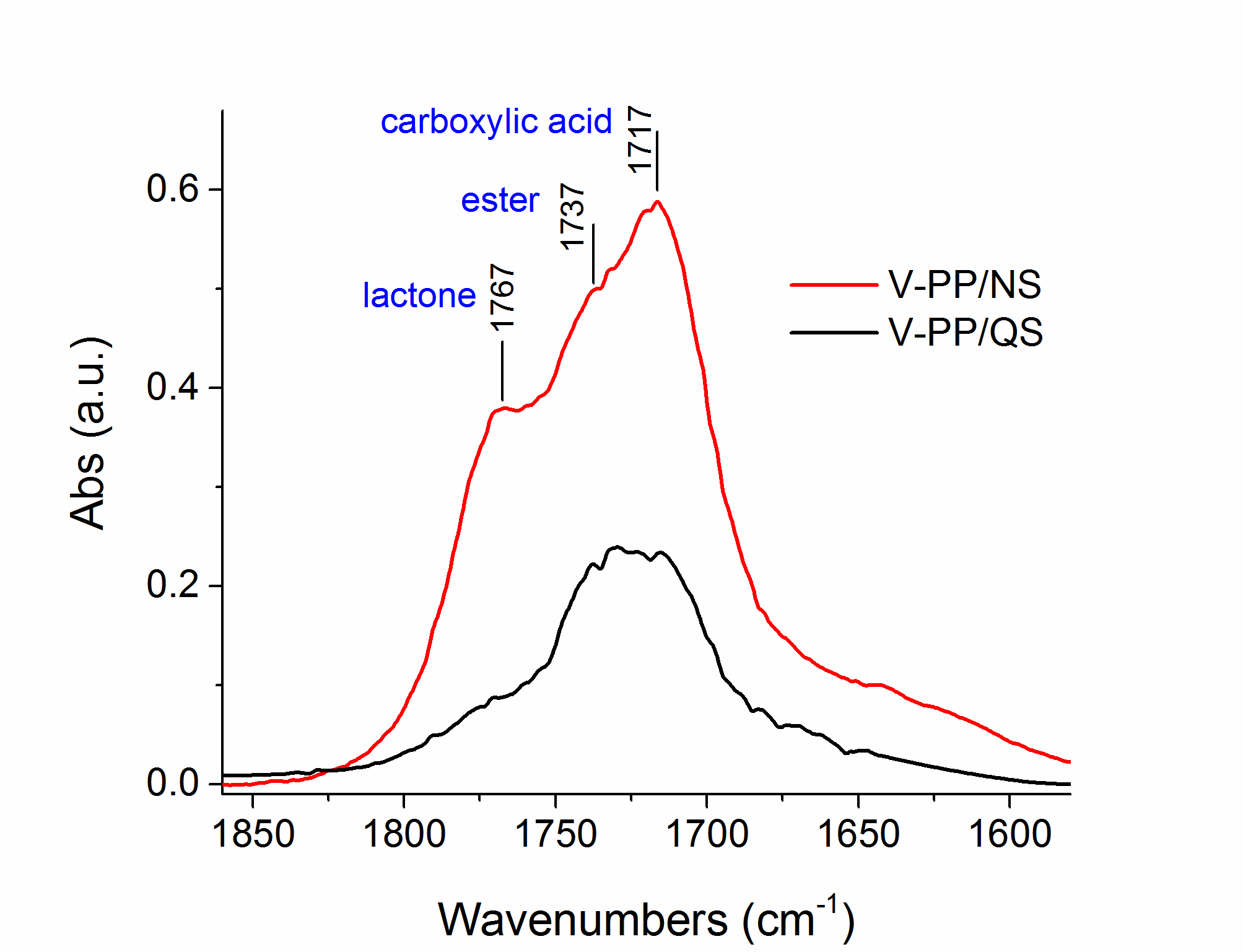


**Figure S2.** Comparison of the carbonyl region in the FT-IR spectra of the DCM extracts from V-PP/QS and V-PP/NS after 20 h UV irradiation (normalized spectra).

The FT-IR spectra of the Xy-extractable fraction (figure S3-S5), typically about 95% of the total polymer even after 20 h UV irradiation, show oxidation levels (carbonyl region around 1700 – 1800 cm^-1^) higher for the V-PP/NS extract than for the V-PP/QS extract.

**Figura S3**. FT-IR spectrum of the Xy extractable fraction of V-PP/NS before irradiation and after DCM extraction.

**Figura S4.** FT-IR spectrum of the Xy extractable fraction of V-PP/NS after 20 h UV irradiation and DCM extraction.

**Figure S5**. FT-IR spectrum of the Xy extract of V-PP/QS after 20 h UV irradiation and DCM extraction.





**Figure S6.** FTIR carbonyl absorptions of the DCM extracts from the Xe-irradiated V-PP/QS_DCM_ and V-PP/QS_DCM_ mixtures, and of the DCM extracts from V-PP before irradiation.


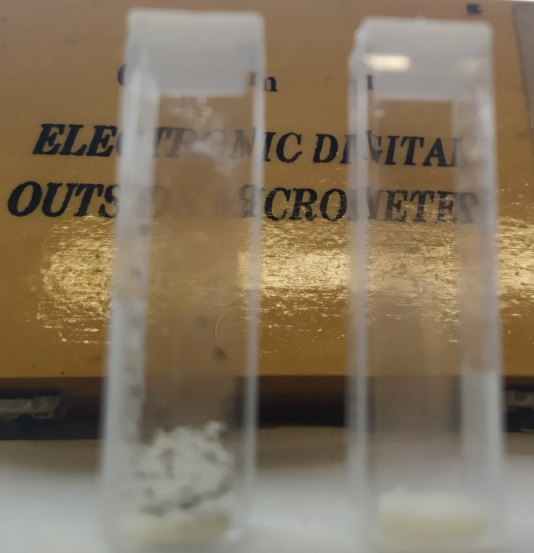


**Figure S7.** Quartz cuvettes containing V-PP with (left) and without (right) iron-containing minerals (NS_Fe_) after 10 days of Xenon lamp irradiation in solar box. The amount of waxy material deposited on the walls of the cuvette as a result of polymer chain fragmentation and sublimation of the newly generated oxidized low oligomers is clearly larger for V-PP/NS_Fe_.

**Figure S8.** SEC chromatograms of the DCM extracts of V-PP exposed to Xenon lamp in solar box in quartz cuvettes in mixture with NS_Fe_ (solid red line) or neat (dashed black line). In the uncalibrated graph the high molecular weight fraction, represented by the peak at lower elution times, is only present in the DCM extract from V-PP/NS_Fe_.

**(a)**

**(b)**

**(c)**

Temperature (°C)

**Figure S9**. Thermogravimetric curves (black line) and first derivative (degradation rate, red line) recorded from the residues of DCM extraction of: a) V-PP; b) V-PP after 35 days of solarbox irradiation; c) V-PP/NS_Fe_ after 35 days of solarbox irradiation (the nearly 40 wt% residue at 600 °C is due to some inorganic NS_Fe_ not completely removed from the sticky oxidized polymer powder).

Weight %

**
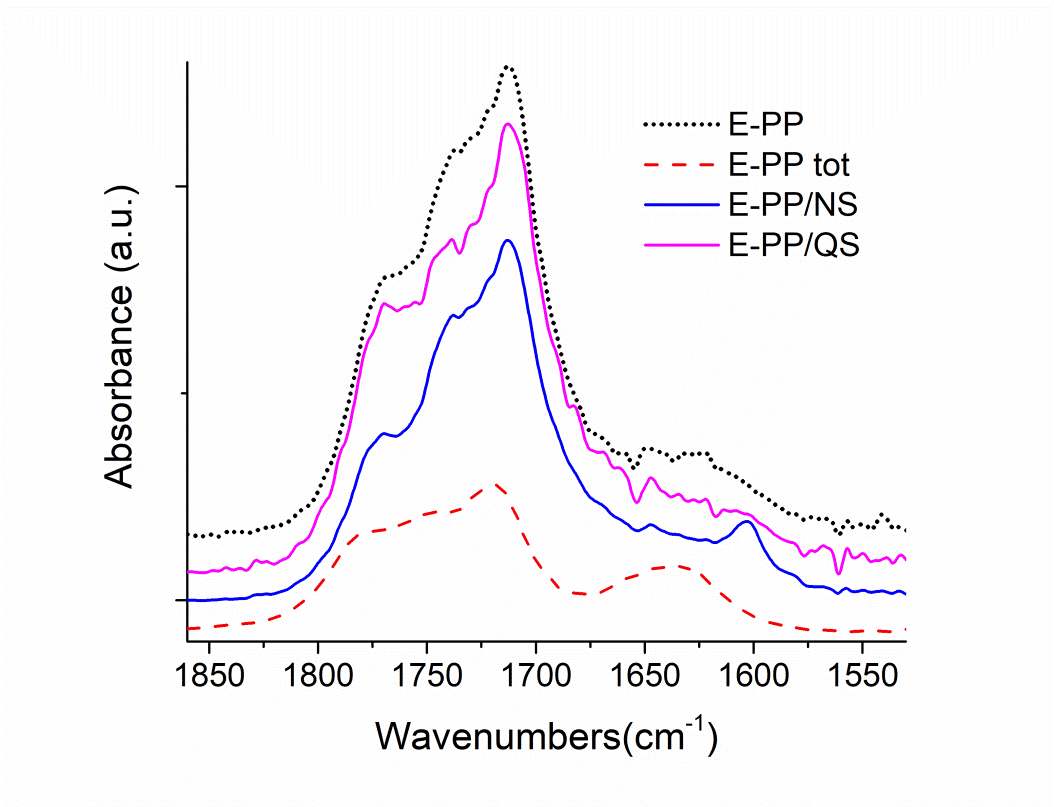
**

**Figure S10**. Carbonyl region in the FT-IR spectra of the DCM extracts of thermally aged E-PP/QS and E-PP/NS; the spectrum of the DCM extract from the unaged E-PP (dotted line) and of the bulk (not extracted) E-PP (dashed line) are also shown for comparison (stacked spectra normalized vs. the methylene bending at 1462 cm^-1^).

**Figure S11**. DCM extracts of the thermally treated E-PP/NS (left) and E-PP/QS mixtures.





**Figure S12**. Normalized FT-IR spectra of the DCM extracts of the thermally aged (35 days) E-PP/QS and E-PP/NS, and of the same fraction recovered from E-PP/NS before thermal ageing. FT-IR spectra of both from E-PP/QS and E-PP/NS mixtures after thermal ageing show the usual structured carbonyl absorption with maxima at 1780, 1769, 1738, and 1713 cm^-1^ from lactone, peroxy acid, ester and carboxylic acid groups

In Figure S13 are reported the FTIR spectra recorded from the xylene extracts of E-PP/QS and E-PP/NS, showing the main ester and carboxylic acids absorptions at 1739 and 1711 cm^-1^.

**Figure S13**. Normalized FT-IR spectra of the xylene soluble fraction (after DCM extraction) of the thermally aged E-PP/QS and E-PP/NS, and of the same fraction recovered from E-PP/NS before thermal ageing. In the inset the detail of the carbonyl absorptions.

**Figure S14.** Comparison of the carbonyl region in the FT-IR spectra polymer separated by flotation from the DCM extracted residue of the thermally aged E-PP/QS and E-PP/NS.
